# Convolutional Neural Network-Based Automated Segmentation of Skeletal Muscle and Subcutaneous Adipose Tissue on Thigh MRI in Muscular Dystrophy Patients

**DOI:** 10.3390/jfmk9030123

**Published:** 2024-07-12

**Authors:** Giacomo Aringhieri, Guja Astrea, Daniela Marfisi, Salvatore Claudio Fanni, Gemma Marinella, Rosa Pasquariello, Giulia Ricci, Francesco Sansone, Martina Sperti, Alessandro Tonacci, Francesca Torri, Sabrina Matà, Gabriele Siciliano, Emanuele Neri, Filippo Maria Santorelli, Raffaele Conte

**Affiliations:** 1 Department of Translational Research and New Technology in Medicine and Surgery, Academic Radiology, University of Pisa, 56126 Pisa, Italy; giacomo.aringhieri@unipi.it (G.A.); emanuele.neri@unipi.it (E.N.); 2 Department of Developmental Neuroscience, IRCCS Fondazione Stella Maris, 56128 Pisa, Italy; guja.astrea@fsm.unipi.it (G.A.); gemma.marinella@fsm.unipi.it (G.M.); rosa.pasquariello@fsm.unipi.it (R.P.); filippo.santorelli@fsm.unipi.it (F.M.S.); 3 Institute of Clinical Physiology, National Research Council of Italy (IFC-CNR), 56124 Pisa, Italy; marfisidaniela@gmail.com (D.M.); francesco.sansone@cnr.it (F.S.); atonacci@ifc.cnr.it (A.T.); raffaele.conte@cnr.it (R.C.); 4 Department of Clinical and Experimental Medicine, University of Pisa, 56126 Pisa, Italy; giulia.ricci@unipi.it (G.R.); francesca.torri94@gmail.com (F.T.); gabriele.siciliano@unipi.it (G.S.); 5 Department of Neurology, Careggi University Hospital, University of Florence, 50134 Florence, Italy; martina.sperti@unifi.it; 6 SOD Neurologia 1, Dipartimento Neuromuscolo-Scheletrico e Degli Organi di Senso, Azienda Ospedaliera Universitaria Careggi, 50134 Florence, Italy; sabrina.mata@aouc.unifi.it

**Keywords:** convolutional neural network, magnetic resonance imaging, automated segmentation, thigh muscles, muscular dystrophies, subcutaneous adipose tissue

## Abstract

We aim to develop a deep learning-based algorithm for automated segmentation of thigh muscles and subcutaneous adipose tissue (SAT) from T1-weighted muscle MRIs from patients affected by muscular dystrophies (MDs). From March 2019 to February 2022, adult and pediatric patients affected by MDs were enrolled from Azienda Ospedaliera Universitaria Pisana, Pisa, Italy (Institution 1) and the IRCCS Stella Maris Foundation, Calambrone-Pisa, Italy (Institution 2), respectively. All patients underwent a bilateral thighs MRI including an axial T1 weighted in- and out-of-phase (dual-echo). Both muscles and SAT were manually and separately segmented on out-of-phase image sets by a radiologist with 6 years of experience in musculoskeletal imaging. A U-Net1 and U-Net3 were built to automatically segment the SAT, all the thigh muscles together and the three muscular compartments separately. The dataset was randomly split into the on train, validation, and test set. The segmentation performance was assessed through the Dice similarity coefficient (DSC). The final cohort included 23 patients. The estimated DSC for U-Net1 was 96.8%, 95.3%, and 95.6% on train, validation, and test set, respectively, while the estimated accuracy for U-Net3 was 94.1%, 92.9%, and 93.9%. Both of the U-Nets achieved a median DSC of 0.95 for SAT segmentation. The U-Net1 and the U-Net3 achieved an optimal agreement with manual segmentation for the automatic segmentation. The so-developed neural networks have the potential to automatically segment thigh muscles and SAT in patients affected by MDs.

## 1. Introduction

Muscular dystrophies (MDs) are a wide group of genetic diseases mainly affecting skeletal muscles and other tissues at variable extent [1]. There is a wide clinical heterogeneity in muscle disorders, even among patients affected by the same disease or even in the same family. In addition, several patients present a slowly progressive, chronic course in which changes in the clinical picture are expected to be seen over a long period of time [2,3].

The genetic characterization can be challenging considering the overlapping phenotypes. Along with these features, the low epidemiological prevalence of rare and ultrarare forms of MDs further complicate the path towards precision diagnosis, outcome measures definition, and design of clinical trials. Given the high social- and health-related economic impact of this group of disorders, it is fundamental to precisely trace the disease progression and response to treatment, especially for children and their families [4]. To date, outcome measures in MDs and congenital myopathies include neuromuscular examination with muscle strength testing, functional motor scales with timed tasks (i.e., Six Minute Walking test, North Star Ambulatory Assessment scale, Performance of the Upper Limb scale, among others), and global physical evaluation, comprehensive of functional cardiac and respiratory assessments. However, based on the upsurge of diagnostic sensitivity and capabilities and surrogate fluid biomarkers tracking disease progression (although with some limitations) [5], there is clear evidence of clinical dissimilarities, prompting further research on the natural history and deep phenotyping of patients. In the past years, muscle MRI has grown as a useful and informative tool in neuromuscular disorders (NMDs), both at a research and clinical level. Several techniques (high magnetic fields, spectroscopy, and semi-quantitative and quantitative MRI) are employed in studying natural history, pathophysiology, and treatment effectiveness [6].

Muscular MRI can provide both qualitative and quantitative information. On the one hand, qualitative muscle MRI is available in many centers and provides clinicians with a reliable instrument for diagnosis and to evaluate disease progression, along with clinical neuromuscular examination and motor functional testing. Indeed, qualitative MRI can easily identify oedema and fatty infiltration (i.e., the main pathologic features), distinguishing between active muscle inflammation and chronic muscle damage, and depicting specific patterns of muscular involvement extensively described in association with various genetic forms [7]. On the other hand, quantitative MRI is mostly used for research purposes to obtain an unbiased measurable evaluation of fatty infiltration (through fat fraction estimation with 2- or 3-point Dixon sequence), oedema (with T2 relaxometry) and early structural damage (via diffusion tensor imaging), and is also potentially useful for evaluating treatment response [8,9,10] As demonstrated in other clinical scenarios, imaging data also contain quantitative information not visible to the naked eye, known as radiomics features, which could be useful for further profiling of these patients [11,12,13,14]. However, extracting quantitative features from specific areas of the image requires manual segmentation, which is extremely time consuming and often subject to variability. Deep learning-based automated approaches could address these issues, allowing for the extraction of radiomics features.

The InGene 2.0 Project, funded by Regione Toscana—Bando Regione Salute 2018, offers an integrated, multiparametric approach in NMDs based on a single software platform, designed to support diagnosis and management of patients towards trials readiness. This occurs through the collection of information at multiple levels, including personal data, neurological examination, phenotypic description, functional motor tests, genetic data, histological evidence of muscle biopsies, and biomedical images. Given the need to better profile patients and improve objective clinical stratification in future treatments, our goal was to develop a deep learning-based algorithm for the automated segmentation of thigh muscles and subcutaneous adipose tissue (SAT) from T1-weighted muscle MRIs from MD patients.

## 2. Materials and Methods

### 2.1. Subjects

This study was approved by the institutional review board of Regione Toscana (CEPR and CEAVNO), and the written informed consent was acquired from each participant.

From March 2019 to February 2022, we retrospectively selected adult and pediatric patients with NMD and in regular follow-up at the Azienda Ospedaliera Universitaria Pisana, Pisa, Italy (Institution 1) and the IRCCS Stella Maris Foundation, Calambrone-Pisa, Italy (Institution 2), respectively.

As inclusion criteria, subjects should have undergone both expert neurologic evaluation suggestive of MDs or congenital myopathy and bilateral thighs MR exam, including dual-echo sequence acquisition. The exclusion criteria were severe motion artifacts, leading to an inadequate image quality and segmentation, and incomplete neurological evaluation.

A total of 23 MD subjects were finally included: 19 patients with unremarkable MR and 4 patients with mild muscular MR abnormalities, meant as fatty infiltration and substitution.

### 2.2. MRI Acquisition

Bilateral thighs MR exams were performed with a 1.5 T scanner (GE Signa HDXT TwinSpeed^®^—software version 15.0, Chicago, IL, USA) and 12-channels body coil, with the following protocol:

- axial T1 weighted in- and out-of-phase (dual-echo);
- axial short-tau inversion recovery (STIR);
- axial diffusion-weighted imaging (DWI) with b values 0 and 500.

The dual-echo sequence technical parameters were TR 120–170 ms, TE 2.5/4.9, flip angle 75°, thickness 10 mm, spacing 2 mm, number of averages 1, and matrix 384 × 352.

### 2.3. Manual Segmentation

Both muscular and SAT were manually and separately segmented on out-of-phase image sets by a radiologist with 6 years of experience in musculoskeletal imaging. The muscles were segmented following the outer margin in both healthy muscles and those with fatty infiltration. Moreover, all the muscles in the thighs were segmented individually and grouped in the anterior (vastus intermedius, vastus lateralis, vastus medialis, rectus femoris, and sartorius muscles), medial (adductor longus, adductor magnus, and gracilis muscles), and posterior (semimembranosus, semitendinosus, and biceps femoris muscles) compartments. The segmentation process was performed using the 3D-Slicer software (Version 4.11).

### 2.4. Image Preprocessing

First, we applied N4 bias field correction [15] to all images in the dataset to account for intensity inhomogeneities. For each subject, the 2D slice at the middle third of the thigh was selected. Subsequently, the left and right thighs were separated to analyze two images per subject (thus yielding a total of 46 images).

The entire dataset was then randomly split in order to obtain training, validation, and test set. In particular, 19 images were used for training, 13 for validation, and 14 for testing. Data augmentation was performed only on the training set by randomly applying horizontal and vertical shifts, horizontal flip, and rotation between −15° and 15°, yielding a total of 380 images for training.

### 2.5. Automatic Segmentation

Two convolutional neural network (CNN) U-Net [16] architectures were trained in Keras with Tensorflow backend on a MacBook Air (macOS Version 11.6) with a 1.8 GHz Intel Core i5 CPU and an Intel HD Graphics 6000 GPU.

U-Nets are a type of convolutional neural network architecture specifically designed for biomedical image segmentation. The “U” in U-Net refers to the symmetric U-shaped struture of the network, which includes a contracting path to capture context and a symmetric expanding path that enables precise localization [14].

Specifically, we first built a U-Net (namely, U-Net1) for the automatic segmentation of all the thigh muscles together (i.e., one output class), as well as the SAT. Then, we built a U-Net (namely, U-Net3) for the automatic segmentation of the three muscular compartments separately (i.e., three output classes) and the SAT.

Both U-Net1 and U-Net3 were trained using a batch size of 380 for a maximum of 300 epochs (early stopping monitoring the cross-entropy loss), with Adam optimizer (learning rate of 10^−5^). Manual segmentations were employed as ground truth to train and validate the U-Nets.

### 2.6. Performance Evaluation

The performance of U-Net1 and U-Net3 was assessed on the test set by comparing manual and automatic segmentations through the Dice similarity coefficient (DSC). The DSC is usually exploited as a measure of the spatial overlap between two segmentations and is defined as

(1) $$DSC=\frac{2TP}{2TP+FP+FN}$$

where TP = true positives, FP = false positives, and FN = false negatives. Specifically, the DSC is the number of overlapping pixels between manual and automatic segmentation divided by the total number of pixels in both segmentations. It ranges between 0 (i.e., no overlap) and 1 (i.e., complete overlap). DSCs for each output class were calculated and the median value was considered.

All performance analyses were carried out by using Python (Version 3.7.3).

## 3. Results

The cohort study included 23 subjects with a mean age of 32.00 ± 16.80 (range 7–58) and was prevalently male sexed (13.56%). Demographic characteristics are shown in Table 1.

The automatic segmentation of the entire test set (i.e., 14 images) took approximately 1 second per image for both U-Net1 and U-Net3. The estimated accuracy for U-Net1 was 96.8%, 95.3%, and 95.6% on train, validation, and test set, respectively, while the estimated accuracy for U-Net3 was 94.1%, 92.9%, and 93.9%.

**Figure 1:**
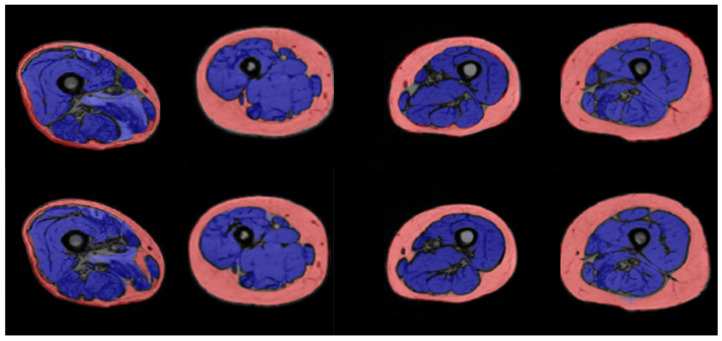
Results of manual (above) and automated (below) segmentation of thigh muscles (blue) and subcutaneous adipose tissue (pink) obtained with the U-Net1 for (from the left to the right) P0, P1, P9, and P12.

**Figure 2:**
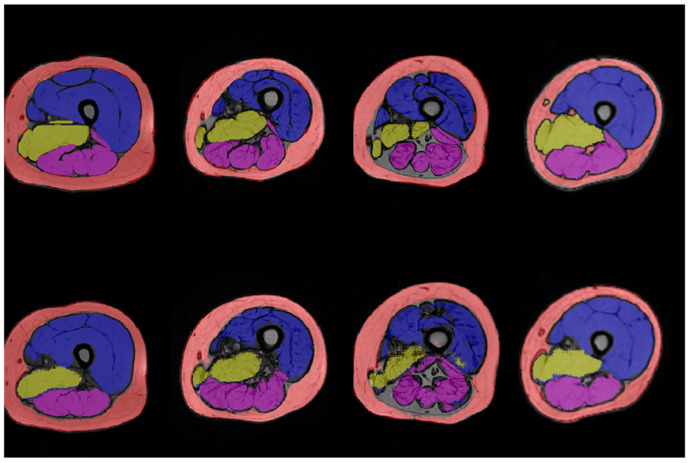
Results of manual (above) and automated (below) segmentation of thigh muscles anterior (blue), medial (yellow), and posterior (purple) compartments and subcutaneous adipose tissue (pink) obtained with the U-Net3 for (from the left to the right) P4, P6, P8, and P10.

Figure 1 and Figure 2 show the results of manual and automatic segmentation obtained with U-Net1 and U-Net3, respectively, for some representative patients. The segmentation of the whole thigh muscle obtained with the U-Net1 showed an excellent qualitative agreement with manual segmentation. A good qualitative agreement could be seen also for the anterior compartment segmented with U-Net3, while the identification of the border between medial and posterior compartments seemed less accurate.

Table 2 summarizes the DSC values computed on the test set for the U-Net1. This quantitative analysis confirmed the excellent agreement between automatic and manual segmentation of the entire thigh muscle, with a median DSC value of 0.95 for both the SAT and the muscle compartment (interquartile range of 0.02 and 0.03, respectively). Analogously, Table 3 summarizes the DSC values computed on the test set for the U-Net3. While the segmentation of the SAT with U-Net3 yielded the same median DSC value as the U-Net1 (i.e., 0.95), the DSC values obtained for the three muscular regions confirmed the qualitative evaluation, with the medial compartment showing the worst performance (i.e., median DSC = 0.7).

## 4. Discussion

In this study, two different CNNs were trained to automatically segment thigh muscles and subcutaneous fat tissues, on axial T1-weighted out-of-phase MR images, yielding an optimal performance.

The U-Net1 and the U-Net3 achieved an optimal agreement with manual segmentation for the automatic segmentation of all the thigh muscles together and the three muscular compartments, respectively. This approach is not only a new tool for research purposes, but it may also help in clinical practice to quantify muscle mass, a relevant yet unmet need when dealing with muscle atrophy, not only in heritable conditions, but also in cancer, inflammatory diseases, sarcopenia, and cachexia [17,18,19,20,21,22].

Similar results were found by Kim et al., who adopted a U-Net transformer architecture to automatically segment individual thigh muscles on computed tomography images and achieved a DSC of 0.84 [23]. While individual muscle segmentation may seem beneficial compared to segmentation of the entire muscle or its compartments, as conducted in our study, it carries computational implications that should not be disregarded. Moreover, the greater number of segmentations resulting from this approach may result in an overly abundant array of quantitative features relative to the patient population size. Indeed, in the same study, grouping of thigh muscles was necessary to demonstrate a negative association with sarcopenia [23].

Moreover, despite the larger availability of CT scans and a common use in the investigation of muscle disorders associated with cachexia or sarcopenia, the added value of qualitative MRI is unquestionable in this clinical setting [24,25]. Even more important is quantitative MRI, given the availability of multiparametric sequence able to capture the wide heterogeneity of NMDs features [26]. Indeed, MR segmentation masks may be easily transferred onto other sequences, enriching the spectrum of quantitative features potentially derived.

Accordingly, several efforts have been made in the literature to train models able to automatically segment lower limb muscles on MRI [27,28,29].

Gaj et al. proposed a DL-based approach to automatically segment thigh muscles compartments and surrounding tissues by combining a U-Net and a Dense-Net [27]. In line with our results, the model had high agreement with manual segmentation (DSC > 0.97).

A compartmental approach was followed also by Okorie et al. and Ding et al. [28,29]. Okorie et al. developed a multi-atlas-based image segmentation framework to automatically segment four functional muscles: gracilis, hamstring, quadriceps femoris, and sartorius muscles of both healthy individuals and patients [28]. As a result, the model achieved a DSC of 0.784, slightly lower compared to our model. The same muscles were automatically segmented by a U-Net, with an average DSC > 0.85 in both individuals and patients, as reported by Ding et al. [29]. In addition, the authors developed a pipeline for reproducibly extracting the fat fraction from the automatic segmentations performed by the CNN.

Agosti et al. adopted a supervised DL approach to segment a greater number of muscles, i.e., twelve and six thigh and leg muscles, respectively, in patients with MDs [30]. Similarly to our model, the authors demonstrated a DSC up to 0.95 with respect to the manually segmented labeled images.

As shown in the aforementioned studies, there is a considerable heterogeneity, not only in technical aspects regarding the model used, but also in the choice of the thigh muscles to be segmented. The compartment-based approach appears to be the most popular, as it represents a valid compromise between the quantity/significance of quantitative features and computational costs. Our work has produced two different neural networks able to provide one and three output classes, respectively, which can be variably combined depending on the requirements. However, to explore the utility of different segmentation approaches, further studies are needed. Within the InGene2.0 project, we are also working on single muscle segmentation as the next step for evaluation. This could be useful to compare the results, usefulness, and practical applicability of the different approaches in the various clinical scenarios of muscular disorders.

In addition, U-Net1 and U-Net3 achieved a median of 0.95 in the segmentation of SAT. The estimation of SAT may provide useful insights in this clinical scenario and in other diseases as well. Several papers have already investigated the potential role of SAT-derived biomarkers (stand alone or in association with other biomarkers), which have been demonstrated to be associated with cardiovascular risk, insulin resistance, and with non-alcoholic fatty liver disease [31,32,33,34,35,36].

Our study has several limitations: first, the retrospective design of the study, which may lead to unforeseen selection bias; second, the small sample size and the use of a limited number of centers to harmonize results; third, the inherently high grade of variability of MDs; and finally, the lack of a reproducibility evaluation and of an external dataset for reference, which may provide useful information about the generalizability of the obtained results.

In conclusion, the so-developed neural networks have the potential to automatically segment thigh muscles and SAT in patients affected by MD.

Further studies are required to assess the reproducibility and to externally validate the neural networks.

## Figures and Tables

**Table 1:** Characteristics of the patients.

**Demographics**	**N (%)**
Age (years, mean ± standard deviation)	32.00 ± 16.80
Male	13 (56)
Female	10 (44)
Caucasian ethnicity	23 (100)
**Diagnosis**	**N (%)**
Becker Muscular Dystrophy	5 (22)
Facioscapulohumeral muscular dystrophy	4 (17)
Spinal muscular atrophy	3 (13)
Limb Girdle Muscular dystrophy (LGMD)	3 (13)
Glycogen Storage Disease Type II	2 (9)
Mitochondrial myopathy	2 (9)
RYR1-related myopathies	2 (9)
Idiopathic hyperCKemia	1 (4)
Duchenne muscular dystrophy	1 (4)

**Table 2:** DSC values computed on the test set for the U-Net1.

	SAT *	Muscle
P0	0.89	0.88
P1	0.97	0.95
P2	0.95	0.97
P3	0.94	0.94
P4	0.95	0.93
P5	0.95	0.95
P6	0.98	0.96
P7	0.93	0.95
P8	0.95	0.94
P9	0.97	0.98
P10	0.91	0.96
P11	0.94	0.95
P12	0.94	0.87
P13	0.88	0.80
Median DSC (IQR)	0.95 (0.02)	0.95 (0.03)

* SAT: Subcutaneous adipose tissue.

**Table 3:** DSC values computed on the test set for the U-Net3.

	SAT *	Anterior	Medial	Posterior
P0	0.88	0.90	0.38	0.01
P1	0.97	0.97	0.30	0.82
P2	0.95	0.96	0.88	0.92
P3	0.94	0.90	0.90	0.95
P4	0.95	0.90	0.74	0.94
P5	0.94	0.90	0.69	0.91
P6	0.98	0.93	0.70	0.91
P7	0.93	0.90	0.90	0.93
P8	0.94	0.86	0.73	0.86
P9	0.97	0.96	0.89	0.91
P10	0.89	0.95	0.92	0.95
P11	0.95	0.94	0.64	0.92
P12	0.95	0.80	0.76	0.77
P13	0.84	0.79	0.36	0.09
Median DSC (IQR)	0.95 (0.02)	0.90 (0.05)	0.7 (0.2)	0.9 (0.1)

* SAT: Subcutaneous adipose tissue.

## Data Availability

The data presented in this study are unavailable due to privacy restrictions.
